# Revealing the unique clinical characteristics of subarachnoid hemorrhage in young adults

**DOI:** 10.1007/s10143-025-03857-8

**Published:** 2025-10-11

**Authors:** Hiroki Kobayashi, Takuma Maeda, Tomoya Kamide, Atsushi Hashio, Akio Teranishi, Yushiro Take, Tomomichi Kayahara, Kaima Suzuki, Hiroki Kurita

**Affiliations:** 1 Department of Cerebrovascular Surgery, Saitama Medical University International Medical Center, Hidaka, Japan; 2 Department of Neurosurgery, Kanazawa University, Kanazawa, Japan

**Keywords:** Subarachnoid hemorrhage, Young adults, Clinical outcomes, Sex differences, Small aneurysms, Hypertension

## Abstract

Subarachnoid hemorrhage (SAH) is a severe condition with high morbidity and mortality rates. While SAH is well-documented in the general population, its characteristics in young adults remain unclear. This study aimed to investigate the specific features of young adults with SAH and to identify predictors of clinical outcomes following SAH in young adults. This retrospective study included 894 patients with aneurysmal SAH who underwent surgical clipping or endovascular treatment between 2012 and 2024. Patients were divided into two groups: the young adult group (< 40 years) and the older adult group (≥ 40 years). Clinical characteristics, aneurysm features, and clinical outcomes were compared between the groups. Among the 894 patients, 42 (4.7%) were classified as young adults. Compared to older adults, young adults had a higher proportion of males (57.1% vs. 30.6%, p < 0.01) and a lower prevalence of hypertension (38.1% vs. 64.2%, p < 0.01). Young adults also had significantly smaller aneurysms than older adults (median: 4.0 mm vs. 5.0 mm, p < 0.01). Both the WFNS (World Federation of Neurosurgical Societies) grade on admission (p < 0.01) and hydrocephalus requiring shunt surgery (p = 0.01) were associated with worse clinical outcomes in the young adult group. Young adults with SAH exhibit distinct clinical characteristics, including male predominance, lower hypertension rates, and smaller aneurysm size. Even in the absence of well-known risk factors, close follow-up and proactive therapeutic interventions are essential for managing intracranial aneurysms in this population.

**Supplementary Information:**

The online version contains supplementary material available at 10.1007/s10143-025-03857-8.

## Introduction

Subarachnoid hemorrhage (SAH) is a severe condition with high morbidity and mortality rates. Survivors often experience permanent disabilities, cognitive decline, and mental health disorders that result in a substantial reduction in their activities of daily living [1]. Perioperative management based on patient age and other characteristics is essential for improving the long-term outcomes of SAH.

SAH incidence increases with age, peaking between the ages of 40 and 60 years [2, 3]. Aging is an independent risk factor for intracranial aneurysm rupture, and the occurrence of SAH in young adults is relatively rare [4, 5]. Although the clinical features of SAH in the general population are well documented, the specific characteristics of SAH in young adults remain unclear. Young adults represent a distinct patient population characterized by unique biological, clinical, and psychosocial attributes, which require tailored management and care [6]. Indeed, there have been reports suggesting that the clinical presentation of SAH in young adults may differ from that observed in the general population [7]. Furthermore, SAH occurrence at a young age leads to high societal costs and substantial loss of productivity, making its prevention particularly important [8]. Therefore, identifying these risk factors is crucial to improve intracranial aneurysm management in this population.

Previous studies on SAH in younger patients have predominantly been limited to single-arm trials with small sample sizes. These studies often vary widely in age range, making direct comparisons difficult. Furthermore, no large-scale comparative studies have been conducted between young adults and older adults with SAH.

In this study, we compared the clinical characteristics and outcomes of SAH between young and older adults to identify unique characteristics and predictors of clinical outcomes following SAH in young adults.

## Materials and methods

**Fig. 1:**
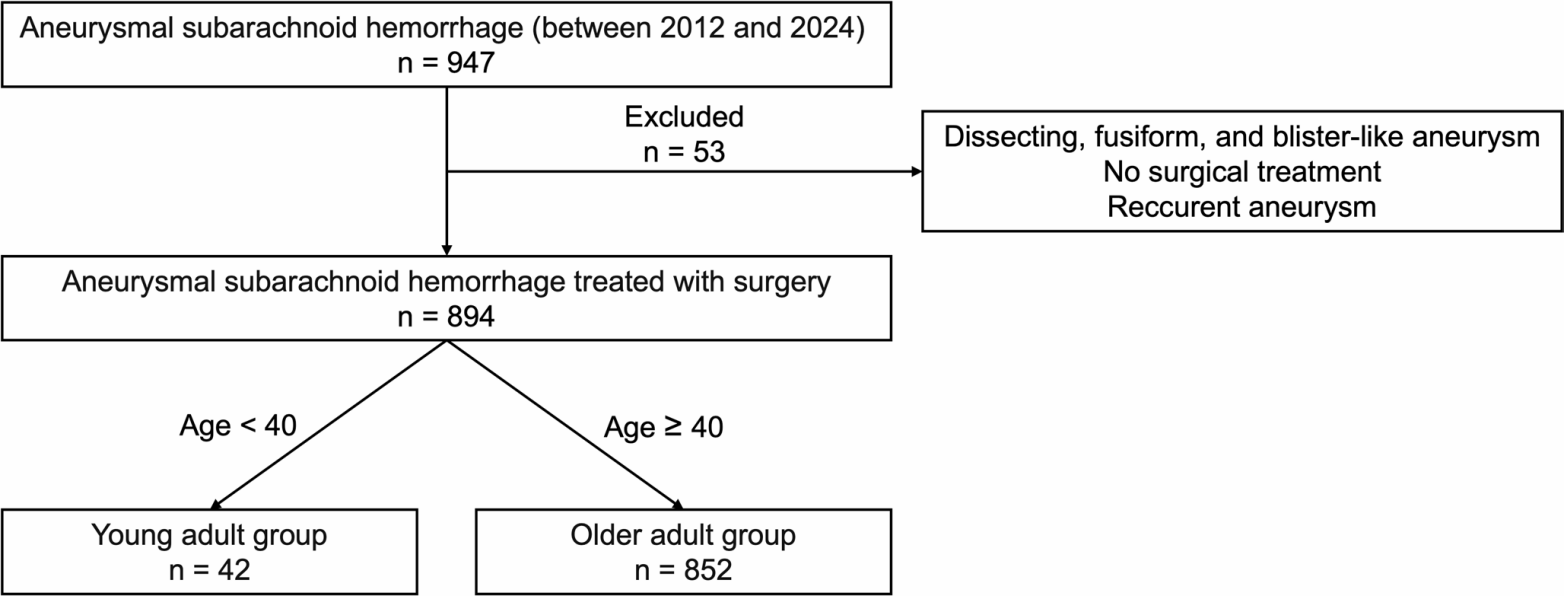
Inclusion criteria

This retrospective study was approved by the institution’s Ethics Committee (approval number: 2024 − 155). Patients were given the opportunity to refuse to participate in the study. This study was based on the current version of the Declaration of Helsinki, and all research procedures were conducted in accordance with the relevant guidelines and regulations. No animal experiments were conducted in this study. This study included 894 consecutive patients with aneurysmal SAH who underwent surgical treatment (surgical clipping or endovascular treatment) at our institution between 2012 and 2024. Fig. 1 depicts a sequence of decisions about study inclusion criteria. This retrospective study was conducted using medical and surgical records. Patients were classified into two groups based on their age at onset: the young adult group (21–39 years) and the older adult group (≥ 40 years). The following variables were compared between the two groups: World Federation of Neurosurgical Societies (WFNS) grade, Fisher group, presence of intraparenchymal hematoma, history of hypertension, family history, sex, aneurysm location and size, therapeutic modality (surgical clipping or endovascular treatment), occurrence of cerebral vasospasm, development of hydrocephalus requiring shunt surgery, and clinical outcomes at discharge according to the modified Rankin Scale (mRS).

The location and size of aneurysms were determined using three-dimensional computed tomographic angiography, magnetic resonance angiography, or digital subtraction angiography. In cases of multiple aneurysms, the ruptured aneurysm was identified based on imaging findings and intraoperative observations. Aneurysm sizes were categorized as follows: small (< 5 mm), medium (5–9.9 mm), large (10–24.9 mm), and giant (≥ 25 mm). SAH cases were limited to those caused by intracranial aneurysms, excluding dissecting and blister-like aneurysms for which size measurement was difficult, cases requiring retreatment, and cases in which surgical intervention could not be performed because of the patient’s systemic condition.

### Statistical analysis

Quantitative variables are expressed as means ± standard deviations. The χ^2^ or Fisher’s exact tests were used to analyze binary categorical variables. For parametric data, unpaired sample *t*-tests with Welch’s correction were performed, whereas Mann–Whitney U tests were used for nonparametric data. Additionally, the multivariable analysis was performed to identify variables predictive of prognosis.

All statistical analyses were performed using SPSS version 29 (IBM Corp., Armonk, NY, USA), with statistical significance set at *p* < 0.05.

## Results

The baseline characteristics of 894 patients are presented in Table 1. The median age was 67.0 years (IQR, 53.0–76.0), with 285 male and 609 female patients (male-to-female ratio = 1:2.1). In total, 392 patients (43.8%) underwent endovascular treatment, and 502 patients (56.2%) underwent surgical clipping. The distribution of the WFNS grades on admission was as follows: grade 1, 260 patients (29.1%); grade 2, 232 patients (26.0%); grade 3, 46 patients (5.1%); grade 4, 157 patients (17.6%); and grade 5, 199 patients (22.3%). The Fisher group distributions were as follows: group 1, 31 patients (3.5%); group 2, 121 patients (13.5%); group 3, 625 patients (70.0%); and group 4, 117 patients (13.1%). Intraparenchymal hematoma was observed in 283 patients (31.7%). A family history was present in 59 patients (6.6%). Among all patients, 63.0% (563 individuals) had a documented history of hypertension. The most frequent aneurysm location was the anterior communicating artery (ACoA; *n* = 252, 28.2%), followed by the internal carotid-posterior communicating artery (ICPC; *n* = 210, 23.5%), middle cerebral artery (MCA; *n* = 201, 22.5%), anterior cerebral artery (ACA; *n* = 62, 6.9%), other internal carotid arteries (ICA; *n* = 61, 6.8%), and posterior circulation (*n* = 108, 12.1%). The median aneurysm size was 5.0 mm (IQR, 3.6-7.0), and the distribution was as follows: small, 394 patients (44.1%); medium, 400 patients (44.7%); large, 96 patients (10.7%); and giant, three patients (0.3%). Symptomatic cerebral vasospasm occurred in 87 patients (9.7%), and hydrocephalus requiring shunt surgery developed in 151 patients (16.9%). Favorable outcomes (mRS score 0–3) were observed in 522 patients (58.4%), whereas unfavorable outcomes (mRS score 4–6) were observed in 372 patients (41.6%).

**Table 1:** Comparison of clinical features and treatment outcomes between young and older adult patients

	All patients	Young adults	Older adults	*p* value*
No. of Patients	894	42	852	
Age (yrs), median (IQR)	67.0 (53.0–76.0)	33.5 (30.0-36.3)	67.0 (55.0–76.0)	
Female sex	609 (68.1)	18 (42.9)	591 (69.4)	< 0.01
WFNS grade				0.13
Grade 1–3	538 (60.2)	30 (71.4)	508 (59.6)	
Grade 4–5	356 (39.8)	12 (28.6)	344 (40.4)	
Fisher group				0.12
1	31 (3.5)	1 (2.4)	30 (3.52)	
2	121 (13.5)	10 (23.8)	111 (13.0)	
3	625 (69.9)	29 (69.0)	596 (70.0)	
4	117 (13.1)	2 (4.8)	115 (13.5)	
Hypertension	563 (60.0)	16 (38.1)	547 (64.2)	< 0.01
Family history	59 (6.6)	2 (4.8)	57 (6.7)	1.00
Location of aneurysm				
ACoA	252 (28.2)	18 (42.9)	234 (27.5)	0.03
ICPC	210 (23.5)	11 (26.2)	199 (23.4)	0.67
MCA	201 (22.5)	6 (14.3)	195 (22.9)	0.19
ACA	62 (6.9)	3 (7.1)	59 (6.9)	1.00
Other ICA	61 (6.8)	0 (0.0)	61 (7.2)	0.11
PC	108 (12.1)	4 (9.5)	104 (12.2)	0.60
Aneurysm size (mm), median (IQR)	5.0 (3.6-7.0)	4.0 (2.7-5.0)	5.0 (3.6-7.0)	< 0.01
Intraparenchymal hematoma	283 (31.7)	8 (19.0)	275 (32.3)	0.07
Therapeutic modality				0.44
Clipping	502 (56.2)	26 (61.9)	476 (55.9)	
Coiling	392 (43.8)	16 (38.1)	376 (44.1)	
Symptomatic vasospasm	87 (9.7)	2 (4.8)	85 (10.0)	0.42
Favorable outcome	522 (58.4)	33 (78.6)	489 (57.4)	0.01
Hydrocephalus requiring shunt	151 (16.9)	3 (7.0)	148 (17.4)	0.08

Values are number (%) except where indicated otherwise. *ACA* anterior cerebral artery, *ACoA* anterior communicating artery, *ICA* internal carotid artery, *ICPC* internal carotid artery–posterior communicating artery, *IQR* interquartile range, *MCA* middle cerebral artery, *PC* posterior circulation, *WFNS* World Federation of Neurosurgical Societies. *Young adults vs. older adults

## Young adults vs. older adults

**Fig. 2:**
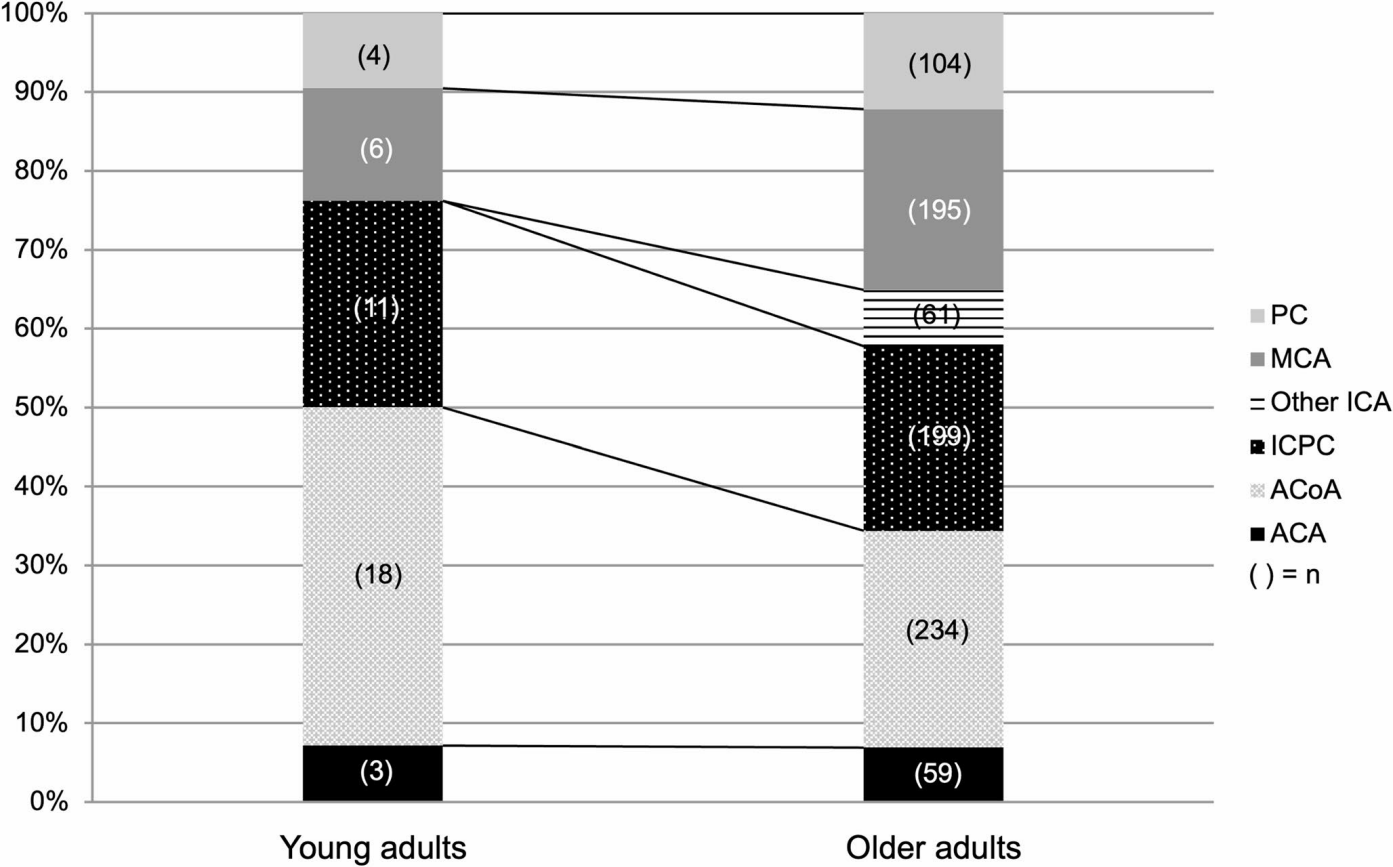
Graph demonstrating the location of aneurysm in patients aged < 40 years and those aged ≥ 40 years. ACA, anterior cerebral artery; ACoA, anterior communicating artery; ICPC, internal carotid artery-posterior communicating artery; MCA, middle cerebral artery; ICA, internal carotid artery; PC, posterior circulation

**Fig. 3:**
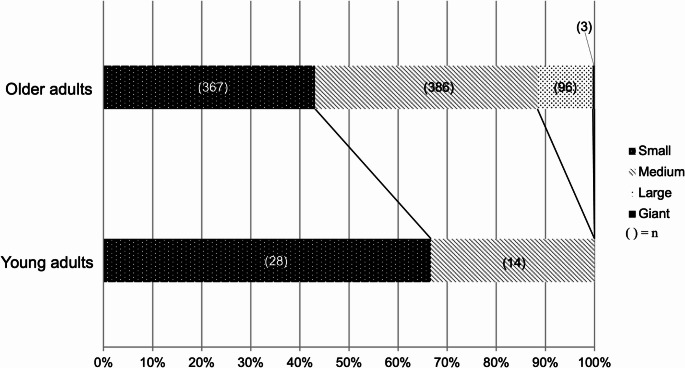
Graph demonstrating the distribution of aneurysm sizes in patients aged < 40 years and those aged ≥ 40 years. Small, < 5 mm; medium, 5–9.9 mm; large, 10–24.9 mm; and giant, ≥ 25 mm

The results of univariate analysis comparing young adults and older adults are shown in Table 1. Among the 894 patients, 42 (4.7%) were classified as young adults and 852 (95.3%) as older adults. The median age in the young adult and older adult groups was 33.5 years (IQR, 30.0-36.3) and 67.0 years (IQR, 55.0–76.0), respectively. The age distribution of all patients, categorized by sex, is shown in Fig. S1. In the young adult group, males (*n* = 24, 57.1%) were predominant, whereas in the older adult group, females (*n* = 591, 69.4%) were predominant (*p* < 0.01). The distribution of aneurysm locations in each age group is illustrated in Figs. 2 and S2. The incidence of ACoA aneurysms was significantly higher in the young adult group than in the older adult group (42.9% vs. 27.5%, *p* = 0.03). Aneurysm sizes in each age group are shown in Figs. 3 and S3. A significant difference in the size of aneurysms was observed between the two groups (median: 4.0 mm vs. 5.0 mm, *p* < 0.01). Fewer young adults had a history of hypertension compared with older adults (38.1% vs. 64.2%, *p* < 0.01). A significant difference in the mRS score at discharge was observed between the two groups; an increased proportion of young adults had favorable outcomes compared with older adults (78.6% vs. 57.4%, *p* = 0.01). Multivariable analysis also showed significant differences in sex (*p* = 0.01), hypertension (*p* < 0.01), aneurysm size (*p* < 0.01), and mRS score at discharge (*p* = 0.03) between the two groups (Table 2).

**Table 2:** Multivariate analysis comparing subarachnoid hemorrhage in young and older adults

	OR (95% CI)	*p* value
Female sex	0.34 (0.18–0.66)	0.01
Hypertension	0.31 (0.16–0.61)	< 0.01
ACoA	1.55 (0.80–3.02)	0.20
Aneurysm size	0.72 (0.60–0.87)	< 0.01
Favorable outcome	2.34 (1.08–5.05)	0.03

*ACoA* anterior communicating artery, *CI* confident interval, *OR* odds ratio

### Predictors of unfavorable outcomes

Univariate analysis was performed separately for the young adult and older adult groups to identify predictors of unfavorable outcomes in each group (Table 3). In the young adult group, the WFNS grade on admission (*p* < 0.01) and hydrocephalus requiring shunt surgery (*p* < 0.01) were predictors of clinical outcomes. In the older adults group, significant differences were observed in age (*p* < 0.01), WFNS grade on admission (*p* < 0.01), Fisher group (*p* < 0.01), family history (*p* = 0.01), aneurysm size (*p* < 0.01), presence of intraparenchymal hematoma (*p* < 0.01), therapeutic modality (*p* = 0.01), symptomatic vasospasm (*p* < 0.01), and hydrocephalus requiring shunt surgery (*p* < 0.01) between the groups. Given the sample size in the older adult group, a multivariable analysis was performed, which identified age (*p* < 0.01), WFNS grade on admission (*p* < 0.01), intraparenchymal hematoma (*p* < 0.01), symptomatic vasospasm (*p* < 0.01), and hydrocephalus requiring shunt surgery (*p* < 0.01) as predictors of poor clinical outcomes (Table S1).

**Table 3:** Comparison of clinical characteristics and treatment outcomes between favorable and unfavorable outcomes in young and older adult patients

	Young adults			Older adults		
	Favorable	Unfavorable	*p* value	Favorable	Unfavorable	*p* value
No. of Patients	33	9		489	363	
Age (yrs), median (IQR)	33.0 (30.0–36.0)	34.0 (29.0-36.5)	0.67	62.0 (51.0–72.0)	74.0 (64.0–81.0)	< 0.01
Female sex	15 (45.5)	3 (33.3)	0.71	339 (69.3)	252 (69.4)	0.98
WFNS grade			< 0.01			< 0.01
Grade 1–3	29 (87.9)	1 (11.1)		399 (81.6)	109 (30.0)	
Grade 4–5	4 (12.1)	8 (88.9)		90 (18.4)	254 (70.0)	
Fisher group			0.20			< 0.01
1	1 (3.0)	0 (0.0)		29 (6.0)	1 (< 0.01)	
2	10 (30.3)	0 (0.0)		90 (18.4)	21 (5.8)	
3	21 (63.6)	8 (88.9)		329 (67.2)	267 (73.6)	
4	1 (3.0)	1 (11.1)		41 (8.4)	74 (20.4)	
Hypertension	14 (42.4)	2 (22.2)	0.44	305 (62.4)	242 (66.7)	0.20
Family history	1 (3.0)	1 (11.1)	0.39	42 (8.6)	15 (4.1)	0.01
Location of aneurysm						
ACoA	14 (42.4)	4 (44.4)	1.00	132 (27.0)	102 (28.1)	0.72
ICPC	9 (27.3)	2 (22.2)	1.00	113 (23.1)	86 (23.7)	0.84
MCA	4 (12.1)	2 (22.2)	1.00	118 (24.1)	77 (21.2)	0.32
ACA	2 (6.1)	1 (11.1)	0.53	39 (8.0)	20 (5.5)	0.16
Other ICA	0 (0.0)	0 (0.0)		33 (6.7)	28 (7.7)	0.59
PC	4 (12.1)	0 (0.0)	0.56	54 (11.0)	50 (13.8)	0.23
Aneurysm size (mm), median (IQR)	4.0 (2.3–5.2)	3.6 (3.1–4.5)	0.86	5.0 (3.5–6.5)	6.0 (3.8-8.0)	< 0.01
Intraparenchymal hematoma	4 (12.1)	4 (44.4)	0.05	90 (18.4)	185 (51.0)	< 0.01
Therapeutic modality			1.00			0.01
Clipping	20 (60.6)	6 (66.7)		255 (52.1)	221 (60.9)	
Coiling	13 (39.4)	3 (33.3)		234 (47.9)	142 (39.1)	
Symptomatic vasospasm	1 (3.0)	1 (11.1)	0.39	40 (8.2)	108 (29.8)	< 0.01
Hydrocephalus requiring shunt	0 (0.0)	3 (33.3)	< 0.01	30 (6.1)	54 (14.9)	< 0.01

Values are number (%) except where indicated otherwise. *ACA* anterior cerebral artery, *ACoA* anterior communicating artery, *ICA* internal carotid artery, *ICPC* internal carotid artery–posterior communicating artery, *IQR* interquartile range, *MCA* middle cerebral artery, *PC* posterior circulation, *WFNS* World Federation of Neurosurgical Societies

## Discussion

Our study revealed that small aneurysms, male sex, and the absence of hypertension were specific characteristics of SAH in young adults compared with older adults. The clinical features of aneurysmal SAH in the young adult population differ from those of general SAH cases, suggesting that commonly used scoring systems such as PHASES may have limitations in predicting aneurysmal rupture in this population.

Although several studies have revealed the clinical characteristics of SAH in younger patients, few studies have compared young adults and older adults. Young adults aged < 40 years represent a distinct patient population characterized by unique biological, clinical, and psychosocial attributes that influence disease manifestation, progression, and treatment outcomes. The young adults often exhibits different disease etiologies and treatment responses compared to pediatric or older adult populations [6]. Recognizing young adults as a separate category is therefore critical for developing tailored diagnostic protocols, treatment strategies, and supportive care interventions that ultimately improve clinical outcomes. This study aimed to reveal the clinical characteristics of aneurysmal SAH in young adults.

When considering all age groups, the risk of SAH is higher in women than in men, and factors such as sex hormones, hypertension, and anatomical or physiological differences are believed to play a role [9, 10]. Endogenous estrogen is important for the normal proliferation and survival of vascular smooth muscle cells, and estrogen deficiency, such as during menopause, increases intracranial artery vulnerability and elevates the risk of SAH [9, 10]. Among younger individuals, the prevalence of hypertension is higher in males than in females [11–13]. While men generally have a higher prevalence of hypertension than women, blood pressure increases sharply in women in their 30 s, and the prevalence of hypertension increases with age, reducing the sex gap in older age [14]. In our study, 40% of young adults with SAH had a history of hypertension, a higher rate than that in the general population but significantly lower than that observed in older adult with SAH. In the young adult group, hypertension was observed in 10 males (41.7%) and 6 females (33.3%), while in the older adult group, it was observed in 166 males (63.6%) and 381 females (64.6%). Although the differences were not statistically significant, there was a tendency for higher hypertension prevalence in males among younger adults, which diminished with age in older adults.

Smoking is a well-known risk factor for SAH, and recent studies have shown that longer smoking cessation periods reduce SAH risk [15]. According to the Ministry of Health, Labour and Welfare in Japan, as of 2021, the prevalence of habitual smoking was 14.8% overall, with 24.8% in men and 6.2% in women. Among men aged 30–40 years, the smoking rate exceeded 30% [16]. Similarly, the World Health Organization’s global data from 2020 indicated smoking rates of 35.5% in men and 7.9% in women, reflecting a higher prevalence in men than in women [17]. In this study, male sex was identified as an independent clinical characteristic of SAH in the young adults group. Several studies have also shown a male predominance in younger patients with SAH [18–20]. Therefore, the high prevalence of hypertension and smoking in young adult men may influence SAH.

In young adults with SAH, factors other than common risk factors, such as hypertension and smoking, have also been suggested to have an influence. Furthermore, with advancing age, the increasing prevalence of hypertension and hormonal changes in women may contribute to a gradual rise in the prevalence of SAH, eventually surpassing that observed in men. Previous studies on young adults with SAH have implicated risk factors such as arterial fragility, metabolic deficiencies, and Type III collagen deficiencies [18–22]. Additionally, a family history of aneurysms predisposes younger individuals to aneurysm formation and rupture due to inherited vascular wall fragility [23]. Although beyond the scope of this study, these non-hypertensive factors may play a substantial role in SAH in the young adult population.

The proportion of ACoA aneurysms was found to be significantly higher in the young adult group. Pediatric and adolescent SAH are commonly associated with anterior circulation, particularly ICA aneurysms [24–27]. In young adults, ACA and ICA aneurysms are reported frequently [19, 28–30]. Park et al. reported that ACA aneurysms were most frequent among young patients, with ACA aneurysms being significantly more common in men and ICA aneurysms being more common in women [19]. Chotai et al. reported that the ACA was the most common location, followed by the ICA and MCA [29]. Horiuchi et al. found that ICA aneurysms were more frequent in patients in their 30s, whereas ACA aneurysms were more frequent in patients in their 40 s [30]. Similarly, Ogungbo et al. reported that the ACoA was the most common site in young adults, followed by the MCA [31]. ICA aneurysms remain prevalent in young adults, similar to pediatric cases; however, ACA aneurysms have also become common. According to Padget, in the anterior circulation, the ICA develops first, followed by the ACA, and finally, the MCA [32]. This developmental sequence suggests that congenital vulnerabilities of the ICA may predispose it to aneurysm formation under hemodynamic stress, whereas ACA aneurysms may require a longer period to develop than ICA lesions. However, ACA aneurysms are known to rupture even when they are small compared with aneurysms at other locations [33, 34]. Ohashi et al. suggested that the high rupture propensity of ACoA and ACA aneurysms may be related to parent vessel diameter and vascular wall thickness [34]. Consequently, although ACA aneurysms may take longer to form than ICA aneurysms, their tendency to rupture at smaller sizes may explain their high frequency in younger patients. In our study, 35 small aneurysms (< 5 mm) were observed in young patients, 18 (52.4%) of which were located in the ACoA.

Another consideration regarding size is that regardless of location, aneurysms in patients aged < 50 years have been reported to carry a risk of rupture, even when the size is small [35, 36]. Ikegami et al. reported that aneurysms < 3 mm were commonly observed in patients aged < 40 years. Similarly, Ohashi et al. demonstrated that although no significant difference in aneurysm size was observed between patients under and over age 40, younger patients tended to have smaller aneurysms than older patients [34]. Sonobe et al. proposed that aneurysms exhibit one of the four distinct growth patterns. Type 1 aneurysms rupture within a short time frame (days to weeks) after formation, whereas type 2 aneurysms grow slowly over several years and rupture during the growth period. In contrast, type 3 aneurysms grow slowly over several years without rupturing, whereas type 4 aneurysms grow to a certain size and remain stable thereafter [36]. Previous studies, including those by Sonobe et al., have suggested that most incidentally discovered aneurysms have already passed the high-risk period and are in the type 4 stage. In contrast, small ruptured aneurysms are likely to belong to the type 1 category and rupture early during their formation [36–38]. Mitchell et al. used mathematical models based on large-scale study data and concluded that the high-risk period for aneurysm rupture may range from 1 to 2 days to approximately 8 weeks after formation [38]. This suggests that type 1 aneurysms are more common in younger patients than in older patients. Ostergaard et al. proposed that while aneurysm formation in the general adult population is primarily influenced by degenerative and hemodynamic factors, congenital medial defects may play an initiating role in younger individuals [25].

In the older adult group, multiple factors contributed to prognosis, whereas in the young adult group, only the WFNS grade on admission and hydrocephalus were predictive. These findings suggest that in the older adult population, poor outcomes are influenced not only by initial neurological damage but also by age-related vulnerability and a variety of complications. Conversely, among the young adult group, only initial neurological damage and the occurrence of hydrocephalus requiring shunt placement significantly impact patient outcomes, underscoring the critical importance of perioperative hydrocephalus management in improving neurological outcomes [39].

### Limitations

This study has several limitations. First, this study was based on retrospective data acquired from a single center. Second, the sample size of the young adult group was comparatively small, which may have compromised the statistical power of this study. Third, ruptured aneurysms were not compared with unruptured aneurysms, making it challenging to identify specific risk factors for rupture. Despite these limitations, we believe our study provides useful preliminary insight into the clinical characteristics and management of intracranial aneurysm in young adult population, while further multicenter and prospective studies are warranted to validate these findings.

## Conclusion

We demonstrated the specific characteristics of young adults with SAH, including a predominance of males, lower prevalence of hypertension, and smaller aneurysm size than older adults. Even in the absence of well-known risk factors for SAH, close follow-up and proactive therapeutic interventions are crucial for managing intracranial aneurysms in the young adult population.

## Supplementary Information

Below is the link to the electronic supplementary material.

Supplementary File1 (DOCX 634 KB)

## Data Availability

The data that support the findings of this study are available from the corresponding author upon reasonable request. This study was conducted retrospectively and did not involve any procedures beyond standard clinical care.
