# Supplementary material for: Revealing the unique clinical characteristics of subarachnoid hemorrhage in young adults
Source: Neurosurg Rev. 2025 Oct 11;48(1):693. doi: 10.1007/s10143-025-03857-8 (PMC12515245; doi:10.1007/s10143-025-03857-8)
Supplement: Supplementary file 1 — Supplementary File1 (DOCX 634 KB) [file 10143_2025_3857_MOESM1_ESM.docx]

**Supplementary information**

**Revealing the Unique Clinical Characteristics of Subarachnoid Hemorrhage in Young Adults.**

Hiroki Kobayashi, MD^1^, Takuma Maeda, MD, PhD^1^, Tomoya Kamide, MD, PhD^2^, Atsushi Hashio, MD^1^, Akio Teranishi, MD, PhD^1^, Yushiro Take, MD, PhD^1^, Tomomichi Kayahara, MD, PhD^1^, Kaima Suzuki, MD, PhD^1^, Hiroki Kurita, MD, PhD^1^.

^1^Department of Cerebrovascular surgery, Saitama Medical University International Medical Center, Hidaka, Japan.

^2^Department of Neurosurgery, Kanazawa University, Kanazawa, Japan


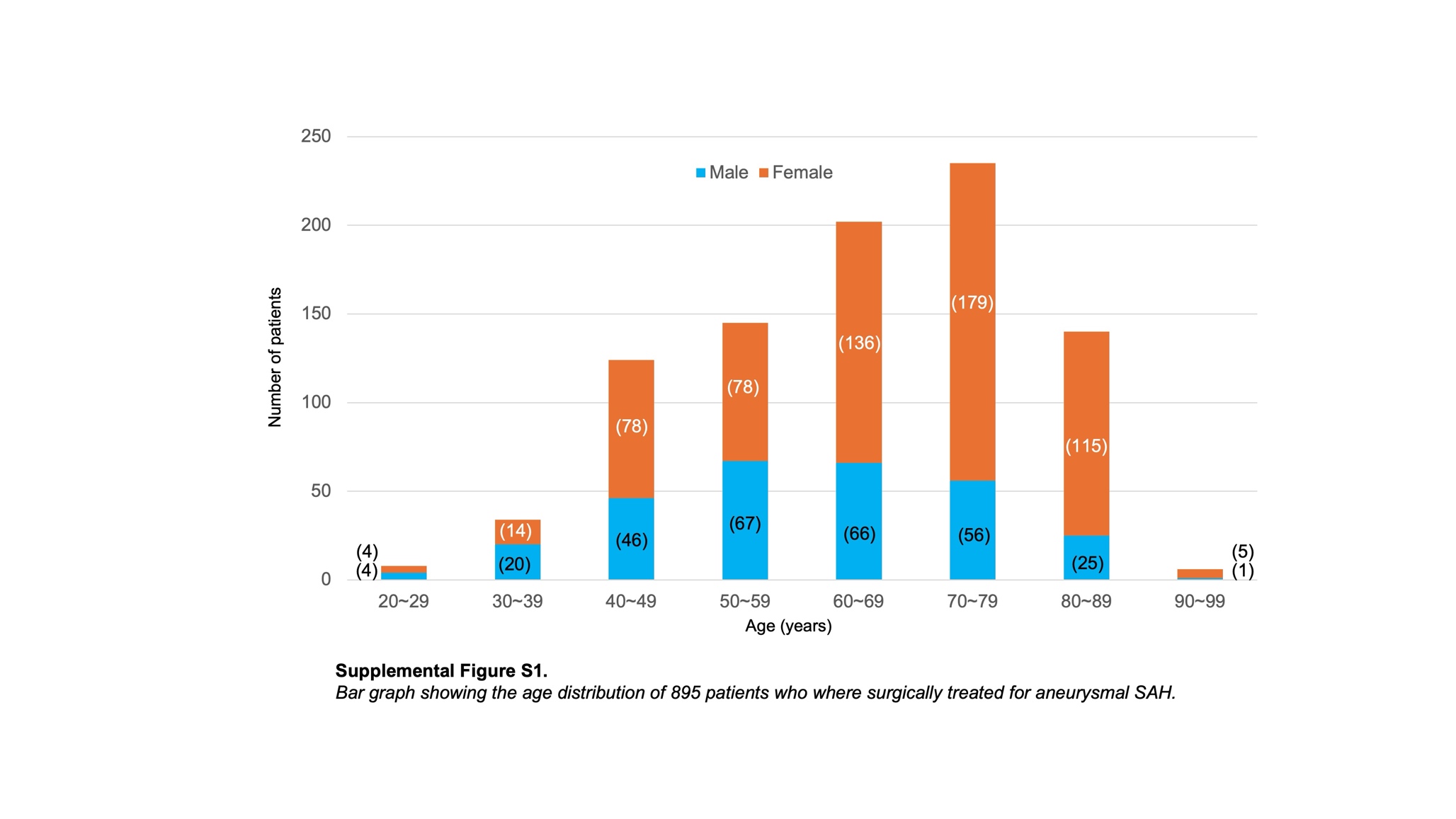


**Supplemental Figure S1**

Graph showing the age distribution of 895 patients who underwent surgical treatment for aneurysmal subarachnoid hemorrhage.


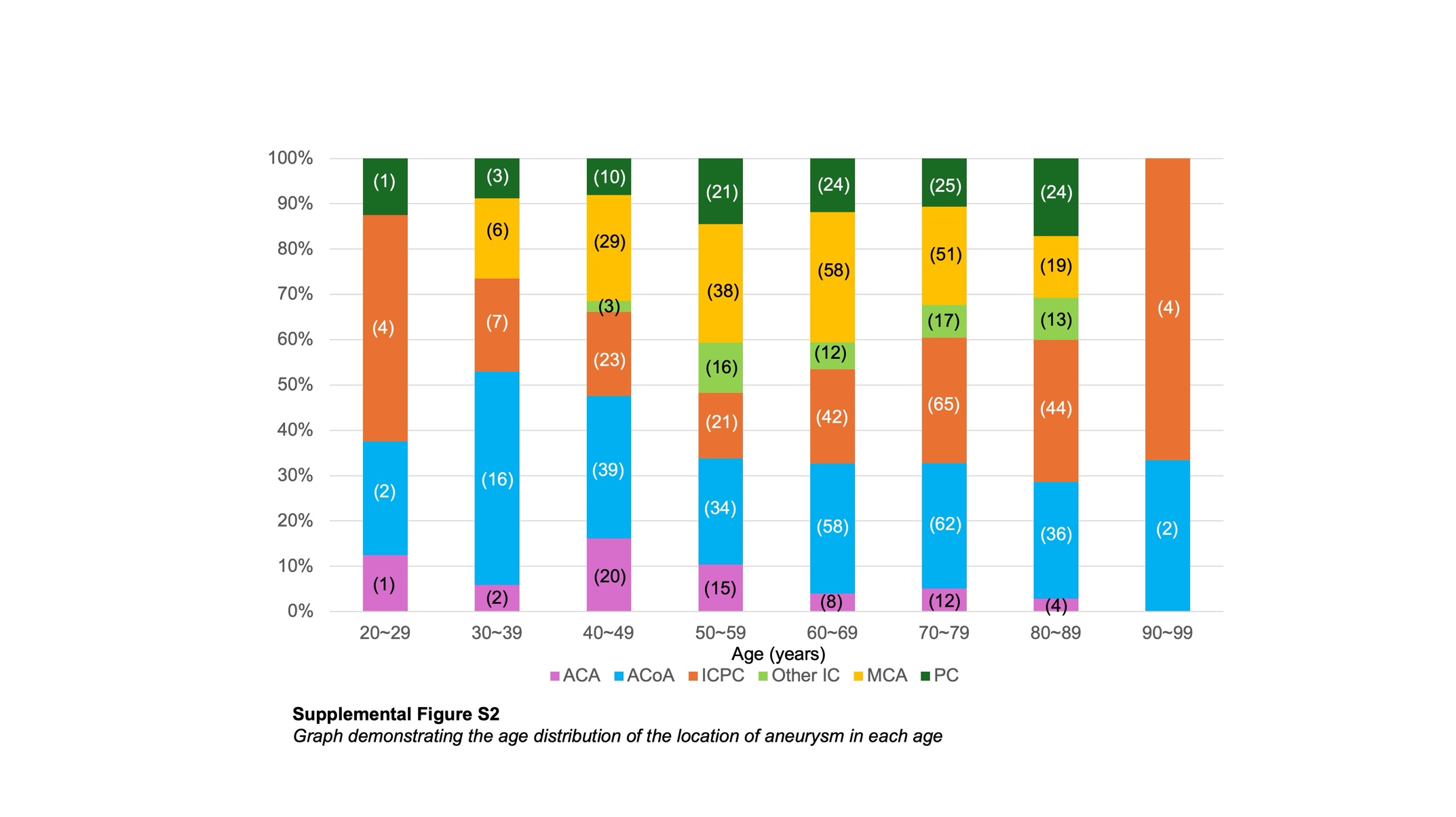


**Supplemental Figure S2**

Graph showing the distribution of aneurysm locations across age groups.


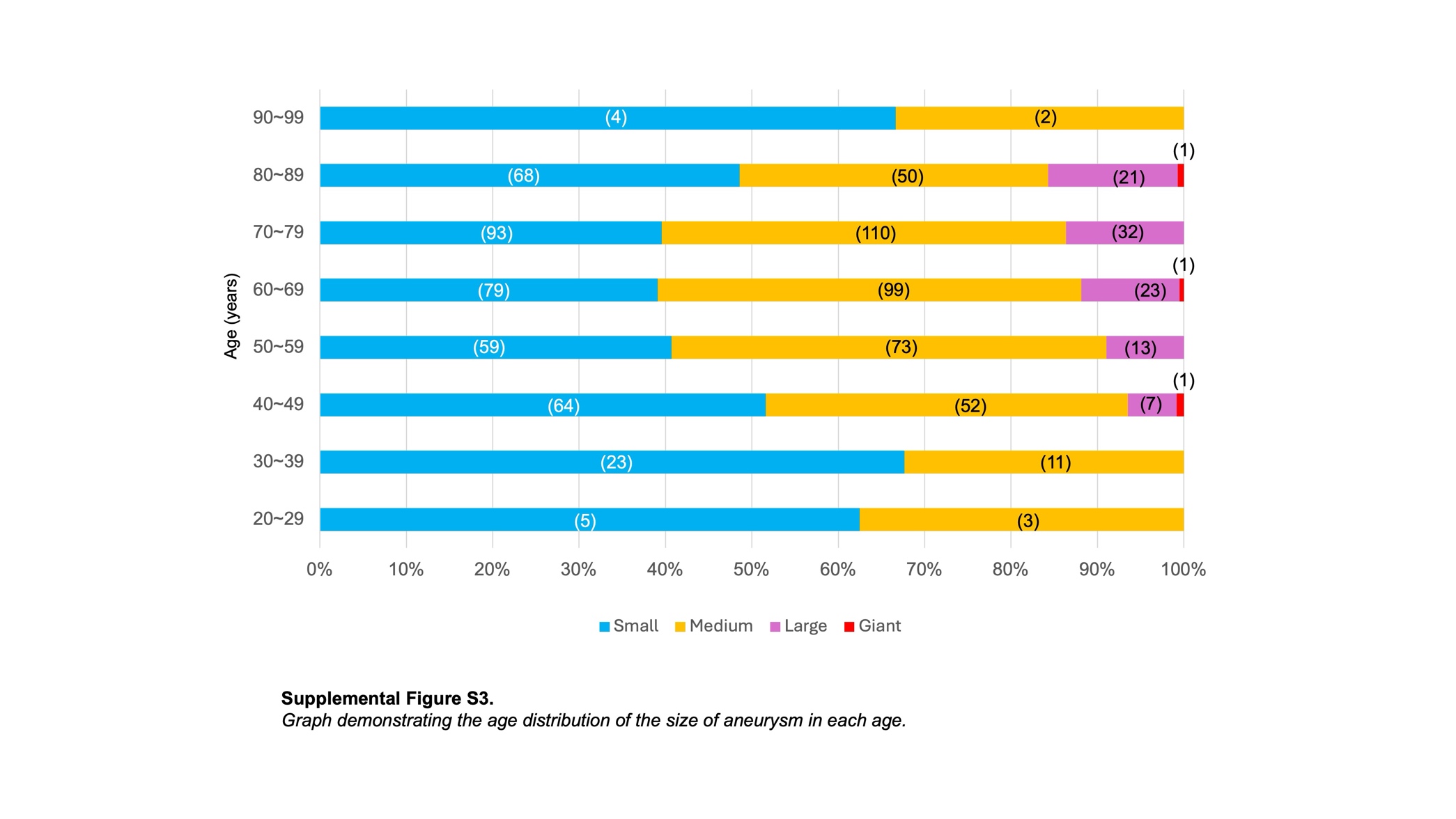


**Supplemental Figure S3**

Graph showing the distribution of aneurysm sizes across age groups.

**Supplemental Table S1.** Multivariate analysis of predictors of unfavorable outcomes in older adults with subarachnoid hemorrhage.

|  | OR (95 % CI) | p value |
| --- | --- | --- |
| Age | 1.07 (1.05 – 1.09) | < 0.01 |
| WFNS grade | 7.06 (4.81 – 10.37) | < 0.01 |
| Family history | 0.77 (0.35 – 1.68) | 0.51 |
| Aneurysm size | 1.04 (0.99 – 1.09) | 0.17 |
| Intraparenchymal hematoma | 2.49 (1.65 – 3.76) | < 0.01 |
| Treatment | 1.06 (0.73 – 1.55) | 0.75 |
| Symptomatic vasospasm | 3.07 (1.68 – 5.62) | < 0.01 |
| Hydrocephalus requiring shunt | 2.94 (1.84 – 4.70) | < 0.01 |

CI, confidence interval; OR, odds ratio; WFNS, World Federation of Neurosurgical Societies.
